# Understanding the Health Literacy Experiences and Practices of Australian-Resettled Myanmar Refugees: Relevance for Nutrition and Dietetics Practice

**DOI:** 10.3390/nu16183109

**Published:** 2024-09-14

**Authors:** Carrie K. Wong, Annie-Claude Lassemillante, Carolynne White, Regina Belski

**Affiliations:** 1 Sport, Performance and Nutrition Research Group, School of Allied Health, Human Services and Sport, La Trobe University, Melbourne, VIC 3086, Australia; a.lassemillante@latrobe.edu.au (A.-C.L.); regina.belski@latrobe.edu.au (R.B.); 2 School of Health Sciences, Swinburne University of Technology, Melbourne, VIC 3122, Australia; 3 Inclusion and Participation, Mind Australia, Burnley, VIC 3121, Australia; carolynne.white@mindaustralia.org.au

**Keywords:** refugee, health literacy, health, Myanmar, service providers, healthcare providers, resettled refugees, healthcare system, nutrition, dietetics

## Abstract

**Background/Objectives:** Refugees typically experience poorer health compared with people from non-refugee backgrounds, and health literacy may play a part in this discrepancy. Using the WHO’s revised health literacy definitions as a framework, this qualitative study sought to examine the health literacy experiences and practices of Australian resettled refugees from Myanmar from refugee and service provider perspectives. **Methods:** Four refugee participant focus groups (*n* = 27) along with one focus group and four interviews with service providers (*n* = 7) were conducted in Melbourne, Australia, and analysed using deductive content analysis. **Results:** Our study found that in addition to individual health literacy, community literacy was practiced by Myanmar refugees, thus highlighting the relevance of social support to health literacy. Furthermore, our study found gaps in healthcare service provision and resourcing related to health literacy development and responsiveness by the healthcare system. **Conclusions:** Our study confirms the relevance of WHO’s revised health literacy definitions to Myanmar refugees while also discussing, in the context of nutrition and dietetics practice, the importance of understanding the different aspects of health literacy and how this relates to working with those who are most marginalised to improve their health and wellbeing.

## 1. Introduction

According to the UNHCR, more than 117.3 million people were displaced globally at the end of 2023, including 1.2 million from Myanmar [1]. The refugee journey can contribute to the poor health of a refugee through insufficient healthcare received in their country of origin or during transition and through interruptions to treatment during the migration journey [2,3]. As a result, many refugees arrive with poorer health in their host country, with health conditions such as diabetes, heart disease, chronic pain, nutrient deficiencies, and mental health disorders commonly reported [4,5,6,7]. Australia has consistently welcomed Myanmar refugees over the past decade, with Myanmar being within the top five countries for humanitarian visas granted [8]. Upon arrival in Australia, humanitarian visa holders are provided with a range of settlement support services, including access to welfare support payments, healthcare, and case management by the Australian government [9,10,11]. In particular, humanitarian visa holders receive access to Australia’s Universal Healthcare System (Medicare), which covers the costs of primary care services, a range of diagnostic tests, health screening, and immunisations. In the State of Victoria, primary healthcare received by resettled refugees often includes allied health, such as dietetics. Despite the availability of comprehensive support services for Australian resettled refugees, they can experience poorer health compared with those from non-refugee backgrounds [12].

Health literacy (HL) may play a role in health outcomes amongst resettled refugees. Health literacy has been causally linked with health outcomes in several ways: people with low HL are more likely to have lower knowledge and understanding of health conditions and their management, are less likely to participate in self-management of their health, are less likely to engage with healthcare providers about their health, and are more likely to visit the hospital, including emergency admissions [13,14,15]. Studies have found that many resettled refugees have low HL [16,17,18], and refugees with low HL are much less likely to seek healthcare compared with those with high HL. This is relevant for nutrition and dietetics as well as for broader healthcare in order to recognise the role of HL being an enabler for health-seeking behaviour.

### Conceptual Understanding of Health Literacy

Early definitions of HL classified it into three cognitive levels: (i) functional/basic, (ii) communicative, and (iii) critical HL, with the focus being on individual skills, capabilities, and the ability to apply these in individual and social contexts [19]. However, there have been calls to recognise HL as more than possession of skills to read and understand health information, but to appreciate the breadth of situations that allow HL to be exercised [20,21]. In 2022, WHO [22] released revised definitions of HL, which includes four facets: individual HL (individual knowledge, confidence, and comfort to engage in daily health activities); community HL (HL assets held by the community to promote health); HL development (the ways in which healthcare systems, providers, and policymakers create enabling environments for healthcare engagement); and HL responsiveness (the extent that the healthcare system recognises the strengths and diverse needs of healthcare users to create environments for healthcare engagement). This is an enhancement from early definitions of HL, which largely only focused on individual attributes of HL and implied that improving HL was an individual responsibility.

A small number of studies have been published on the health literacy of refugees either in a host country or a country of transit, and these have found that refugees generally possess inadequate or low HL [17,18,23]. There is therefore a gap in understanding of the health literacy of resettled refugees, including in the Australian context. Considering that people from refugee backgrounds experience greater health inequities than those from non-refugee backgrounds, understanding how they practice and experience HL has implications for nutrition and dietetics practice. Therefore, our study sought to understand how HL is practiced and experienced amongst Australian resettled refugees from Myanmar. In particular, we were interested in examining HL using the WHO revised definitions and thereby exploring whether these aligned with the HL experiences of resettled refugees from Myanmar. To do so, we examined the perspectives of Myanmar refugees as well as service providers to gain an understanding of views and insights that relate to HL practice as well as the system that supports these.

## 2. Materials and Methods

### 2.1. Study Design

This was a qualitative study as we were interested in gaining a more in-depth understanding of participants and their experiences [24], and we employed a social constructivist paradigm of inqury whereby we recognise that reality is constructed through social interactions [25]. At the time of the study, all authors were healthcare professionals by background but worked as university academics. None identified as being a refugee or from Myanmar as an ethnic background. However, CKW previously worked as a dietitian and had two years of experience working with Myanmar refugees in Melbourne. Ethics approval was obtained from the Swinburne University of Technology Human Research Ethics Committee (2017/119).

### 2.2. Setting and Participants

The study was conducted in several suburbs in Melbourne, Australia, between August 2018 and November 2019. The detailed community participatory approach and methods of this study have been previously published [26]. In summary, recruitment material, data collection, and interpretation of results were conducted in partnership with a steering committee comprising the research team and Myanmar community leaders. Participants included resettled refugees from Myanmar and service providers who provided services in these suburbs. Refugee participants were recruited from a government-funded English language program (ELP) and were invited to participate if they were from Myanmar as country of origin, aged between 18 and 65 years. Exclusion criteria included anyone who was born in Australia or was not from Myanmar as a country of origin, those aged under 18 years or over 65 years, and those who were unable to give informed consent due to mental or cognitive impairment. ELP participants are those who had arrived in Australia within 2 years and aged at least 18 years old. ELP participants reflected migration trends from Myanmar, where the age of migrants was predominantly 25–54 years. Prior to data collection and after obtaining consent from the teachers, two authors (AL, CW) attempted to build rapport and trust with students over a period of three weeks by attending the English language classes in person, chatting with students, and discussing the purpose of the research and what participation would involve. Service providers who worked with Myanmar refugees were recruited using purposive sampling. Service providers were identified based on CKW’s knowledge of the field as well as recommendations from service provider participants. Inclusion criteria included service providers from government-funded or not-for-profit organisations who provided any form of assistance to Myanmar refugees related to health or wellbeing. Exclusion criteria included service providers who did not provide any services to Myanmar refugees, people who provided help and assistance to refugees from Myanmar on a voluntary basis, or those who were unable to give informed consent. CKW previously worked with refugees for around two years; therefore, some rapport and trust were already established with service providers, which potentially helped with recruitment. Nonetheless, CKW no longer worked in the healthcare sector at the time of the study and therefore did not have any direct influence over the work of service provider participants. The research team clearly explained that participation in the study was voluntary, data collection would be anonymous, and other members of the research team could conduct the service provider focus groups or interviews instead of CKW if participants preferred. All service provider participants agreed to be interviewed by CKW.

### 2.3. Data Collection and Analysis

Four focus groups were conducted with refugee participants (*n* = 27) by AL and CW, and one focus group and four individual interviews with service provider participants (seven participants in total) were conducted by CKW. Focus groups were held with refugee participants at the same location as their English language classes to minimise inconvenience. The authors sought permission from the teachers of the English language classes to take participants out of classes for a maximum of two hours to attend interviews. Interpreters matching the preferred languages of students were provided for all focus groups, and refugee participants were invited to respond in either English or in their preferred language via interpreters. Interviews or focus groups were held with service providers at their workplace for their convenience and based on their availability. Prior to the commencement of each focus group or interview, verbal consent was obtained from participants, followed by the completion of a short demographic survey. A topic guide was used for focus groups and interviews for both participant groups (Table 1). All authors exercised relationality [27] by being open, empathetic, and affirming the challenges reported by refugee participants in managing their health, as well as challenges experienced by service providers in providing services for refugees. As the authors did not identify as being from Myanmar, they did not want to make any assumptions that we understood Myanmar culture or practices. Therefore, the authors always sought to clarify meanings and used paraphrasing as a means of checking with participants that their responses were interpreted accurately. In service provider interviews and focus groups, CKW asked all service provider participants to share their insights as if CKW had no prior understanding, so as to reduce assumptions. CKW reflected key points of discussion. as a means of checking that what was said had been interpreted accurately. Focus groups and interviews were audio-recorded using a digital audio recorder. Notes were taken throughout, documenting observations of body language and mannerisms and key insights that prompted further exploration in each focus group and interview. These field notes were also used for comparison, context, and triangulation during data analysis. At the conclusion of focus groups/interviews, key points were summarised and reported back to participants as a means of member checking to ensure that they had been correctly interpreted. Member checking was also conducted with the steering committee, and translated terms were verified for meaning; for example, ‘heavy heart’ was confirmed as an alternative to ‘poor mental health’ as there is not an equivalent term in some Myanmar languages. Refugee participants were provided with a gift voucher for their participation. Audio-recordings of refugee focus groups were transcribed verbatim by AL and CW, while service provider focus groups and interviews were transcribed verbatim by a transcription company. Transcripts were checked three times for accuracy by the researchers as well as to familiarise them with the data.

Using the WHO’s revised definitions of health literacy [22] as a framework, interview transcripts were analysed using deductive content analysis [28]. Deductive content analysis can be used to explore how/if a framework is aligned and the degree of alignment [29]. Main categories were determined based on the framework, with key categories correlating to the four HL areas according to the WHO HL definitions. Coding rules were determined for codes [30], such as coding an entire sentence or statement when seeking examples of HL relevant to the main categories to account for the context by which the statement was made; data that did not fit under the main categories was not coded. Interview transcripts and codes were checked numerous times to ensure data was coded correctly against main categories. An analysis of the data was undertaken to examine how it aligned with the different aspects of HL under the framework and its overall relevance to the practice of HL. Authors referred to their field notes and post-interview reflections for insights that provided context for data.

## 3. Results

Twenty-seven refugee participants aged 18 years and over were recruited (12 men and 15 women). Participants spoke a range of languages, including Burmese, Hakha Chin, Karen, and Zomi. At the time of data collection, nine participants had lived in Australia for less than two years, while 18 had lived in Australia for more than two years (Table 2).

A total of seven ‘front-line’ service provider participants who worked with refugees of all ages in a range of service areas were interviewed (Table 3). One of the seven participants interviewed was both a service provider and identified as being from a refugee background and a member of the Myanmar community, thus providing valuable dual insights.

### 3.1. Knowledge, Where It Comes from, and Links with Health

Health was regarded as important amongst refugee participants, and across all refugee focus groups, participants shared many examples of actively engaging in practical behaviours for their physical and mental health. Healthy eating and nutrition were one of these.

*“To study about food. What’s healthy and not healthy. [To] get the ideas of what to cook and not to cook… education about food and nutrition, what kind of food is good for our body, for example, what kind is good for our eyes, what kind of food is good for our… brain and thing in our body, education.”*

[Refugee FG 2]

Another participant also echoed this sentiment; however, their desire to learn more about food and nutrition appeared to stem from a lack of overall knowledge, the perceived importance of healthy eating, as well as a desire to learn from qualified health professionals, e.g., a dietitian.

*“Yeah we need education, because yeah dietitian and information, what to eat, what not to eat, yeah we need more information about healthy food… we don’t really know what is healthy eating sometime we know that fruits is good sometime we eat two or three at the same time and vegetables a lot of the time. So we might amount and how many times a week and some information would be very helpful…That’s why I’m talking about the education. We should know about, if you educated ok you understand what you are talking, what you are writing we get it that’s why it is very important.”*

[Refugee FG 3]

It was evident that while health was regarded as important for Myanmar refugees there were gaps in their knowledge, and they wanted further help from credible sources to bridge this gap and support them to self-manage their health.

Service providers also reported gaps in health knowledge amongst Myanmar refugees, and attributed this to reasons they did not engage in health-promoting behaviours such as healthy eating and physical activity. In fact, most did not name many examples of how Myanmar refugees actively participated in their health, despite being asked the question *“What do they [refugees from Myanmar] do to look after their health/wellbeing?”* in interviews. Service providers highlighted that knowledge gaps may be due to a lack of education, which influenced the ability of Myanmar refugees to discuss health:

*“I’d probably tie that up with education to a degree as well…but they can give lots of different physical symptoms that don’t seem to make a lot of sense because they put it all together and think I’ve got this really sore knee but it’s related to my shoulder…”*

[Service Provider interview 2, three SPs]

In particular, the extent of health knowledge varied depending on the refugee journey of transition, hence influencing their overall understanding of health as well as how to communicate it with health professionals.

*“I think it makes a difference in terms of the schooling that they’ve had… And it also makes a difference if they’ve gone to a transit country like Malaysia and depending on how long they’ve been there for. Sometimes you can have a teenager that’s been in Malaysia for four, five, six years where they’ve got a bit of English and so their idea of health and how they’ve feeling is different. As well, because they’ve actually been in contact with more health providers than if they were in a camp or a village.”*

[Service Provider Interview 2, three SPs]

However, one healthcare service provider also noted that Myanmar refugees may have different conceptualisations of health compared with Western medicine, and this was reflected in the way in which health was experienced and expressed.

*“They’ll say feeling hot inside and then a lot of the time we transcribe that to say a fever, whereas I think a lot of the time that’s not so much what they’re actually experiencing. Because they really say it’s feeling hot on the inside, rather than feeling fevers and chills like we would describe it.”*

[Service Provider interview 3, one SP]

While knowledge is a key part of individual health literacy, our study shows that the acquisition, interpretation, and translation of knowledge relating to the understanding of health and health behaviour amongst Myanmar refugees is complex and multi-faceted.

### 3.2. Social Networks and Their Role in Community Literacy and Supporting Health Behaviour

Social networks, which consisted of family, friends, the church, and the broader Myanmar community, played a significant role in providing Myanmar refugees with support for their health post arrival. For example, physical activity was more likely to be performed together with social networks rather than alone.

*“I play handball and basketball, sometimes football with my cousins, brothers, sister.”*

[Refugee FG 1]

One service provider concurred with this, however, adding that the social participation of physical activity was situation-dependent.

*“I think that for the women, often they’re socially isolated so they’re not engaged in a lot of physical activity, they might have young children which makes that very difficult. Sometimes they’re not engaging in social activity, particularly if they’re not part of the church.”*

[Service Provider Interview 3, one SP]

The social nature of health participation amongst Myanmar refugees had benefits for physical health but also allowed the application of collective cultural knowledge to continue health practices with other community members after their arrival in Australia. One service provider described how Myanmar refugees engaged in vegetable growing, which they previously performed in their country of origin.

*“They used to all come there with one of my volunteers, and they had a big veggie patch, and then that became a second veggie patch, and so on. And as they were comfortable going there on their own, they actually hired their own veggie patches, so they’re still doing that now.”*

[Service Provider interview 5, one SP]

Collective knowledge sharing was also an important means of disseminating crucial health information in the broader Myanmar community, thus was regarded as a strength. In particular, community leaders such as church pastors were relied upon by Myanmar refugees as trusted sources of information and support about health.

*“The pastors or some of the other members of the church can provide good health [information/advice] if there’s problems or distress or they’re just having problem trying to navigate the Australian system. So, yes, definitely, their communities are a great source of help for their social wellbeing.”*

[Service Provider interview 1, one SP]

In fact, community leaders saw it as their responsibility for helping Myanmar refugees navigate the healthcare system, as well as due to the high trust in them from their community:

*“And even for the leadership level in the community, they need to have more I think training or support levels, or awareness of mental health issues, and get a referral system to service providers at a professional level. Because many people in the community rely much on community leaders, especially religious leaders that they trust in the community.”*

[Service Provider interview 4, one SP]

At the same time, the reliance on community leaders for health information and support also stemmed from challenges experienced by Myanmar refugees with managing their health, such as communication barriers. Therefore, community leaders ‘stepped in’ to mediate these challenges.

*“Yes, especially for people who can’t speak English by themselves. There’s no way that they can do it. And people rely on their community leaders, especially on their pastor. So, now the community leaders are taking up that role to support because there is no one else to do that.”*

[Service Provider interview 4, one SP]

For resettled refugees from Myanmar, social support networks are therefore integral for the communication, sharing, and dissemination of health knowledge, as well as for supporting health behaviour.

### 3.3. The Healthcare System as a Health Enabling and Health Literacy Responsive Environment–Trustworthy, but Not Always Easy to Access or Use

Attributes of the Australian healthcare system can facilitate healthcare access for Myanmar refugees. For example, one refugee participant described the financial support they received to access healthcare in Australia as an enabler:

*“[Health] was difficult to get in a refugee camp… as growing up in a poor country we can’t afford to buy good food, nutrition whatsoever and even when we are sick we cannot easily go to the doctors since we do not have enough money. But arriving in Australia we can buy anything new [we] want easily and the government help us to support financially and so we can, when we are sick we can easily go to doctor. So it’s good a lot of change happen.”*

[Refugee FG 1]

A service provider agreed with this sentiment and further added that the comprehensiveness of Australian healthcare contributed to trust and confidence to use healthcare services:

*“People see that the healthcare system in Australia is a very good system actually, because from the second country or the first country, from Burma (Myanmar), people have to pay money. But here, everything is free except for particular maybe diseases. And people cannot believe that the healthcare system in Australia is so good.”*

[Service Provider interview 4, one SP]

Despite the high trust in Australian healthcare, aspects of the Australian healthcare system also made it challenging for refugees to access it when and where needed. Participants reported that the Australian healthcare system appeared to be fragmented, was difficult to understand, and navigate. For example, healthcare appointments were not always in the same location and were challenging for refugees to get to since they relied on public transport.

*“And sometimes with the time really limited, that [we] have to go from one place to another, it is really hard for [us] to go to on time sometimes.”*

[Refugee FG 3]

Furthermore, healthcare services were not always responsive to the healthcare needs of refugees, as they were often unable to access healthcare when needed, as one refugee participant articulated:

*“Sometimes when we get sick and then make an appointment and we can’t get the same date so wait another two or three days.”*

[Refugee FG 3]

Another service provider added that delays in healthcare had negative impacts on health:

*“So the fact that a lot of our patients have to go through the public system, so they have to wait a long time for outpatient appointments. Some medical issues go unseen and untreated because they don’t have the money to pay for private and the public health system is just overloaded with some specialties. That can be quite difficult.”*

[Service Provider interview 3, one SP]

Healthcare access policies did not always account for the difficulties of healthcare access experienced by refugees, including financial costs and communication barriers with HCPs. One service provider provided an example of this inflexibility in healthcare access:

*“Some clinics now have the policy that they’ll see them … but if they don’t turn up for their appointments…they charge…these people can’t afford to be charged $70 if they don’t turn up for an appointment…. they can’t afford to go if they’ve got to pay.”*

[Service Provider interview 2, three SPs]

To help Myanmar refugees communicate with HCPs and receive information about health, the use of interpreters was integral in appointments because they often had limited English proficiency. Whilst interpreters could be arranged by health services, this was not always a consistent practice. In particular, one service provider explained the consequence of this inconsistency and reliance on family members to convey information by some health services on the healthcare for a young Myanmar child:

*“We had one patient, it was a child and their surgery was cancelled. And when we asked the patient’s parents why they asked for the surgery to be cancelled, and they said no, we didn’t ask for that, we want our child to have the surgery, and the hospital’s adamant that they called the dad and spoken to the dad on the phone. And I asked the dad and the dad said no. The dad didn’t speak in English. And the hospital said they hadn’t used an interpreter. And I was like, you couldn’t have spoken to the dad. It must have been someone else. And they were like, well… They said he was the dad. And I think that the problem was they didn’t use an interpreter to start with, they just called using English. And then luckily, because they’ve made the mistake, they’re able to rebook the surgery. But initially, they told us he needed to be referred through the clinic and it would take a whole lot of six months. It was only when, I think I called them and explained the situation they were very helpful in the end.”*

[Service Provider interview 3, one SP]

There is a need for healthcare providers to be more understanding of the health needs of refugees from Myanmar, the difficult life circumstances they experience, and to provide more empathetic healthcare. When asked, one service provider participant recommended:

*“Maybe more knowledge of their journey, to health providers, like in hospitals, would help as well because I have heard a little bit of negative stuff there where some of our [refugee clients] have had to go in through emergency, and the way they’ve been spoken to.”*

[Service Provider interview 5, one SP]

Another added the need for healthcare providers to be more empathetic to the challenges experienced by Myanmar refugees to attend appointments.

*“I think some understanding around the fact that either they might not have known that they had the appointment or they might not have known how to change it or cancel it, or even just trying to get public transport. If you translate, if your bus is late, you can’t really help that. A bit more flexibility around appointments.”*

[Service Provider interview 3, one SP]

Although there are positive attributes of the Australian healthcare system that make healthcare trustworthy and are appreciated by Myanmar refugees, equally, study participants reported attributes of the healthcare system that make it challenging, even difficult, for Myanmar refugees to obtain professional health support when needed. The responsibility for navigating and accessing the healthcare system appeared to be the responsibility of Myanmar refugees; however, certain actions can be taken by healthcare providers to build confidence and trust in refugees and to better support them in accessing healthcare.

## 4. Discussion

This study explored the health literacy experiences and practices among Myanmar refugees by examining refugee and service provider viewpoints using the amended WHO HL definitions as a framework. Utilisation of both perspectives revealed rich findings related to the WHO HL facets of individual and community literacy, health literacy development, and responsiveness with relevance to nutrition and dietetics practice.

At an individual literacy level, both refugee and service provider participants regarded knowledge as being closely linked with health behaviour amongst Myanmar refugees, including the importance of nutrition knowledge for good health. Knowledge about health varied among refugee participants and was impacted by their refugee journey, where they resided during transition, and the resources that were available during transition. For instance, those who spent most of their time in transition in refugee camps tended to have lower schooling and access to healthcare compared with those who lived in Malaysia during transition. In this study, participants attributed a lack of health knowledge to lack of formal education or lack of healthcare prior to settlement in Australia. However, we acknowledge that some health messages are shared in some refugee camps, for example, those on the Thai-Myanmar border where there has been a presence of foreign non-government aid or healthcare organisations [31]. The need for Myanmar refugees to increase/improve knowledge was expressed by both refugee and service provider participants. Refugee participants in our study gave significantly more examples of actively engaging in different health behaviours, such as eating healthy, compared with service providers. This gap between refugee and service provider perspectives may at least in part be attributable to the deficit-based lens we often see used in western healthcare [32], which does not recognise the strengths and assets that refugees can bring to improve their health. Similarly, such a deficit’s based lens when regarding patient health literacy has previously been reported amongst dietetics practitioners [33]. Nonetheless, people from refugee backgrounds have been demonstrated to not only naturally value health but also possess problem-solving abilities to overcome barriers encountered in their new home country and act for their own health if empowered to do so [34]. Hence, we encourage nutrition and dietetics practitioners and other HCPs to identify strengths and reinforce health-enhancing behaviours undertaken by refugees and recognise these as positive demonstrations of survival and self-empowerment. HCPs should also appreciate that what is regarded as ‘knowledge’ may differ from that of typical western health conceptualisations. Whilst religion/spirituality and health are not normally associated together in western medicine concepts, they are closely linked in Myanmar culture [34,35,36]. For example, one concept of medicine (*hsay*) in Myanmar culture has Buddhist influences. It includes everything that has the power (*swan*) to prevent or cure misfortunes and illnesses believed to be caused by an imbalance of the key elements air, water, earth, and fire [37]. Hence, medicinal practice seeks to restore balance and involves a combination of remedies from natural ingredients, chanting Buddhist scriptures, use of diagrams, etc. Given that many Myanmar refugees still value cultural conceptualisations of health and related health practices such as traditional medicine and foods, these should be honoured and respected as part of appreciating their knowledge and skills in contributing to individual HL [31]. Further, HL should be viewed holistically beyond individual attributes and skills. In contrast to individual literacy, we found many examples of community literacy in our study, with health being regarded by Myanmar refugees as a collective effort and linked with community [38]. As found in this study and in others [39], some health behaviours were practiced together with other community members, such as growing vegetables and doing team sports. This collectivist approach to health is commonly found in many non-West cultures [40,41,42] and refugee groups [11,43,44]. Consequently, when working with diverse cultures, health literacy should be regarded as more than an individualistic responsibility. Community leaders were found to play particularly significant roles in health information sharing amongst the Myanmar community. This is a common occurrence in minority groups in Australia, particularly where existing health resources are not responsive enough or sufficient to meet community needs. For instance, during COVID-19 in Australia, community leaders took on the responsibility of translating and sharing vital information about health measures with their communities, resulting in rapid dissemination [45,46]. As a result, communities were able to follow recommended prevention measures promptly, something that would not have been possible if information were disseminated through traditional channels, which would create lengthy delays. Additionally, community leaders are commonly regarded with high trust amongst minority communities, hence making them preferred sources of information when access to health professionals is not available and during times of crisis [47,48,49]. The significant contributions of community leaders should therefore be acknowledged, as they provide many voluntary hours to fill resource gaps. On the other hand, this could also be considered a disproportionate burden on community leaders, with such reliance on community leaders to disseminate health information leading to a blurring of professional boundaries as well as a risk of burnout [46,50]. Given that HCPs and policymakers are responsible for health resource distribution, there should be improved resource allocations to support community leaders to meet the needs of their communities.

Healthcare organisations and settings are responsible for health literacy development and responsiveness, thus play important roles in creating a supportive health literacy environment for healthcare users [51]. Still, although the healthcare system is generally well regarded by refugees, the existing healthcare system structure and design create numerous challenges for service access by Myanmar refugees. Our study revealed numerous systemic barriers that impeded timely health service access, including rigid service access criteria or policies, long waiting lists for appointments, and insufficient use of interpreters, which fail to mediate language barriers. Such findings align with those from other studies about healthcare barriers amongst Myanmar refugees [31,34,52] and other refugees from different ethnic origins in Australia and abroad [53,54,55,56]. Interestingly, service provider participants were much more likely to report on and provide details about service access barriers experienced by Myanmar refugees, particularly negative experiences. It is possible that this is due to the courteous and polite nature of Myanmar refugees, as well as their general gratitude for healthcare they received, particularly when comparing with pre-settlement [57]. Nonetheless, this does not mean that refugees do not experience some form of discrimination or marginalisation when attempting to use the healthcare system [52,58]. In fact, it is possible that despite significant challenges, they may not feel empowered or safe to voice their concerns. This becomes an issue of uneven power distribution between HCPs, the healthcare system, and healthcare users. Whilst the Australian healthcare system may appear to be comprehensive and universal, it is structured in a way that assumes everyone uses it in the same way regardless of background or social status. Healthcare systems such as those in Australia and the USA assume the ability for patients to access services such as travel to healthcare settings to attend appointments, the ability to navigate healthcare systems, and some proficiency in English. However, as reported in this study as well as others [52,59,60], refugees often experience multiple challenges with healthcare access. There is a need for changes to be made to improve healthcare access and ensure equity, i.e., considering who might be ‘left behind’. Some suggested areas of change include utilising strength-based and community-based approaches to healthcare, greater flexibility in health service responses, and greater understanding of HCPs to the refugee journey as well as culture in their discussion and response to refugee health issues [61].

This study has some limitations. As only Myanmar refugee participants from one part of Melbourne (Australia) were included, not all Myanmar ethnic groups were represented in this study. Nonetheless, as refugee participants represented various Australian Myanmar groups and provided common themes around health, the findings are still useful in helping us understand the health enablers and challenges experienced post-settlement. While researchers attempted to encourage elaboration of responses from all participants, service provider participants were much more eloquent and extensive in their responses to questions compared with refugee participants; therefore, there may be incomplete views expressed by refugees that were not captured in our interviews. In particular, some service providers had worked with Myanmar refugees for extended periods of time and therefore may have built strong trust, which had led to refugees openly sharing their healthcare experiences with them. Although we attempted to build rapport and trust with refugee participants, we acknowledge that this was over a short period of time due to study constraints. If there was prolonged engagement with refugee participants and deeper trust was built, there may be a possibility of greater story-sharing of health experiences from refugees that could provide even richer insights. Additionally, we recognise that the focus group environment may have been less conducive to comprehensive discussions and sharing by individuals compared with interviews. We also acknowledge that there may have been some responder bias from service provider participants, as some of the recruited service providers previously worked with CKW. Therefore, our service provider data may lack some additional perspectives. It is also possible that some service providers may have withheld some information because they knew CKW. Nonetheless, our findings show that in many cases, service providers provided comprehensive responses and stories that revealed significant insights.

## 5. Conclusions

Overall, our study found congruence between the WHO HL definitions and HL experiences and practices of Australian resettled refugees from Myanmar, particularly in the areas of individual and community literacy. Some examples of health literacy development and responsiveness related to refugee experiences of healthcare were identified; however, we recognise that these were more comprehensively named by service providers rather than refugees. Further research into HL development and responsiveness of healthcare providers to the HL needs of resettled refugees is required to gain a more comprehensive picture of HL gaps and potential areas of improvement. Additionally, further research into the understanding and application of HL principles based on current HL definitions in nutrition and dietetics practice can also help the profession better address current gaps in service delivery and create a more positive health literacy environment for service users, especially those who are the most marginalised.

## Figures and Tables

**Table 1:** Interview Guide.

*Interview Guide Questions*	
Refugee Participants	Service Provider Participants
From your experience, what does health mean to you? Why?	From your experience, what does health mean to refugees from Myanmar? Why?
From your experience, what does well-being mean to you? Why?	From your experience, what does well-being mean to refugees from Myanmar? Why?
What do you do to look after your health?	What do they do to look after their health?
What do you do to look after your well-being?	What do they do to look after their well-being?
From your point of view, what do you think is an ideal healthy lifestyle in Australia? Why?	From your point of view, what do you think for refugees from Myanmar is an ideal healthy lifestyle in Australia? Why?
What do you think makes it hard/challenging for you to live a healthy lifestyle in Australia?	What do you think makes it hard/challenging for refugees from Myanmar to live a healthy lifestyle in Australia?
What do you think helps/makes it easier for you to live a healthy lifestyle in Australia?	What do you think helps/makes it easier for refugees from Myanmar to live a healthy lifestyle in Australia?
	What do you/does your organisation do to help refugees from Myanmar live a healthy lifestyle in Australia? How do you decide what service/program to offer to meet their need?

**Table 2:** Refugee Participant characteristics.

	Men (*n* = 12)	Women (*n* = 15)
	*n* (%)	*n* (%)
**Age**		
18–25	2 (16.7)	6 (40)
26–35	1 (8.3)	2 (13.3)
36–45	5 (41.7)	1 (6.7)
≥46	4 (33.3)	6 (46.7)
**No. of Years Lived in Australia**		
≤2 yrs	4 (33.3)	5 (33.3)
>2 yrs	8 (66.7)	10 (66.7)
**Martial status**		
Married	9 (75)	11 (73.3)
Not married	3 (25)	4 (26.7)
**No. of People Living in Household**		
1–2	1 (8.3)	0 (0)
3–4	2 (16.7)	5 (33.3)
5–6	6 (50)	3 (20)
>6	3 (25)	7 (46.7)

**Table 3:** Service provider participant characteristics.

	*n* (%)
**Gender**	
Male	1 (14.2)
Female	5 (71.4)
No data provided	1 (14.2)
**From a Refugee Background**	
Yes	1 (14.3)
No	6 (85.7)
No data provided	1 (14.2)
**Length of Service at Present Organisation**	
<7	0 (0.0) 4 (57.1)
>7 yrs	4 (57.1) 2 (28.6)
No data provided	1 (14.2)
**Length of Time Working with Refugees**	
<2 yrs	0 (0.0)
2–7 yrs	2 (28.4)
7–10	2 (28.4)
>10 yrs	2 (28.4)
No data provided	1 (14.2)
**Services Provided to Refugees**	
Health	6 (85.7)
Education	2 (28.4)
Case Management	1 (14.2)
Social services including housing and employment	1 (14.2)
Faith based services	1 (14.2)

## Data Availability

Data are not available due to ethical restrictions.
